# Chamomile in combating SARS-Cov-2

**DOI:** 10.6026/9732063002002045

**Published:** 2024-12-31

**Authors:** Ahmed M. Tolah, Lamya M. Ahmed

**Affiliations:** 1 Department of Medical Laboratory Technology, Faculty of Applied Medical Science, King Abdul-Aziz University, Rabigh, Saudi Arabia; 2 Special Infectious Agents Unit, King Fahd Medical Research Center, King Abdulaziz University, Jeddah, Saudi Arabia

**Keywords:** Chamomile, COVID-19, SARS-CoV-2, antiviral activity, inhibition

## Abstract

Traditional herbal medicine is of known history for the complementary treatment of viral infections and was recently suggested for COVID-19. Therefore, it is of interest to investigate chamomile decoction for its neutralizing activity against SARS-CoV-2 *In vitro*. Our experiments highlight the potential antiviral effect of chamomile. *In vitro* results show a significant inhibition of SARS-CoV-2. Our results recommend the use of chamomile as a potential natural remedy for COVID-19.

## Background:

In late December 2019, an outbreak of pneumonia of unknown cause began in Wuhan, Hubei Province, China, and has rapidly spread worldwide [1]. Chinese researchers discovered a previously unknown Beta coronavirus [2], which was named Severe Acute Respiratory Syndrome Coronavirus 2 (SARS-CoV-2), caused a disease called COVID-19 that can be transmitted from person to person [3 and 4]. The SARS-CoV-2 symptoms include persistent cough, breath shortness, loss of taste and smell, and fever. In severe cases, the infection leads to secondary bacterial pneumonia, dyspnoea, kidney failure, and even death [5]. SARS-CoV-2 is a positive sense RNA virus, belongs to the Beta coronavirus genus of the Coronaviridae family [6]. The genome of SARS-CoV-2 is composed of fourteen open reading frames that encode four structural proteins, sixteen non-structural proteins, and several accessory proteins [7]. Viral fusion to the host cell endosome is promoted by the cellular surface serine protease. Because of alarming levels of spread and severity, COVID-19 was declared a public health emergency of international concern on January 30th, 2020, and situated as a pandemic on March 2020 by World Health Organization (WHO) [8]. For more than two years, SARS-CoV-2 is still a global threat, especially with the newly evolved variants. By the end of October 2022, the number of confirmed SARS-CoV-2 cases worldwide was more than 628 million, with more than 6.5 million reported deaths [9]. Since the SARS-CoV-2 pandemic declaration, research groups worldwide have prepared themselves to face the problem. Now they have successfully developed a number of effective vaccines [10]. Although approximately 13 billion vaccine doses have been administrated [10], cases and deaths are still rising.

Since the start of SARS-CoV-2 pandemic, researchers have strived to find and discover antiviral medications against SARS-CoV-2 that prevent the spread of the virus [11]. Natural products have demonstrated strength in the discovery pipeline for diverse anti-infective agents, including antiviral drugs. Natural products have shown their potential against several SARS-CoV-2 targets and are considered a precious trove of drug leads [13, 14 and 15]. In the past two years, various natural products (e.g., baicalin, ivermectin, and artemisinin) were reported as promising SARS-CoV-2 inhibitors. They target multiple viral and host-specific proteins involved in viral processing (viral protease), viral entry into host cells, viral replication, and finally, viral release from infected cells [12]. For example, ivermectin is an FDA-approved anti-parasitic natural product isolated from Streptomyces avermitilis that was among the first reported anti-SARS CoV-2 agents and has also demonstrated antiviral efficacy in clinical trials [16, 17]. Even though, The FDA has not approved its use in preventing or treating COVID-19 in humans. Recently, a group of plant-derived phenolic compounds has shown potent *in vitro* anti-SARS CoV-2 activity via targeting the viral main protease Mpro [18, 19]. The medically important plants containing specific phytoconstituents could provide a wide scope as therapeutic against COVID-19. Numerous docking simulations studies have recommended using these compounds to improve COVID-19 therapy. These phytoconstituents represent a promising option for treating coronavirus infection by targeting viral protein and inhibiting viral replication or endocytosis [20].

In this study, we have screened multi plant decoctions for the discovery of new antiviral natural therapeutics against COVID-19. Only chamomile flower (Matricaria chamomilla) decoction showed promising potential antiviral effect against COVID-19 and inhibited the viral replication *in vitro*. Chamomile is a medicinal plant that is widespread in Asia, Africa, and Europe. Chamomile has been used thousands of years ago as a natural remedy for different illnesses. Germany's Commission E approved chamomile to be used as herbal tea bag, drops, capsules, tablets, or liquid extract for the treatment of common cold symptoms [21]. Flavonoids are among the most necessary metabolites in chamomile that have been reported to possess antiviral activity [22]. Inhibition of protease 3c is the direct mediated of flavonoids against coronaviruses [22]. The protease 3c is essential for SARS-CO-V-2 replication and would be a promising drug target [22]. In a cross-sectional study survey, 80.2% of a total of 1,747 participants in Peru indicated that they used medicinal plants to prevent or treat COVID-19 [23]. It is of interest to show, we have conducted an extensive in silico-based investigation by utilizing all the currently available and well-characterized viral protein targets to determine the main constituents responsible for the antiviral activity of chamomile and to show their *in vitro* activity.

## Materials & Methods:

## Preparation of the crude extract:

Crude chamomile flower (Matricaria chamomilla) powder was purchased from herbal stores and authenticated by the botany department, King Abdulaziz University, Jeddah, Saudi Arabia. Then, the powder was used in the experiment without any purification. Crude chamomile powder was boiled in aqueous Dulbecco's Modified Eagle Medium (DMEM) 10% at a concentration of 6.75 mg/mL (w/v), vortex 15 second than incubated for 15 minutes at room temperature and the final volume of the extract obtained was 7 ml, fluid was subsequently sterile filtered using 0.2 mm sterile filter to obtain an aqueous chamomile extract or what is called chamomile decoction. As a primary and rapid screening, we first tested this extract for their cellular cytotoxicity. Non-toxic extract concentrations were then screened for their viral inhibitory activity.

## *in vitro* antiviral assay:

## Virus and cells:

Vero E6 cells (ATCC® number 1568) were maintained and grown in Dulbecco's Eagle medium (DMEM) contained 10% fetal bovine serum (FBS) [24]. SARS-CoV-2/human/SAU/85791C/2020 (Gene accession number MT630432.1) was isolated from a human nasopharyngeal swab confirmed positive by RT-PCR. IRP number H-02-K-076-00520-298 was obtained from the Saudi Ministry of Health to use patient samples. All experiments involved live SARS-CoV-2 were performed following the international recommended safety measures and precautions in Biosafety Level 3 Facility at the Special Infectious Agent Unit, King Fahd Medical Research Center, King Abdulaziz University, Saudi Arabia. SARS-CoV-2 was propagated and titrat using Median Tissue Culture Infectious Dose (TCID50). In brief, SARS-CoV-2 was inoculated on 90%-95% confluent Vero E6 cells in a T175 tissue culture flask and incubated at 37°C for 1 hour in a humidified 5% CO2 incubator with shaking every 15 min. Then, 25 mL of viral inoculation medium (DMEM supplemented with 10 mmol/L HEPES, 1% streptomycin and penicillin and 2% FBS) was used to replace the inoculum. The cells were then incubated at 37°C in a humidified 5% CO2 incubator for 72 hours or until 90% of the cells illustrated CPE (cytopathic effect). The supernatant was then harvested and centrifuged at 500 x g for 5 min at room temperature. Ultimately, SARS-CoV-2 was aliquoted and stored at -80°C and the plaque assay was used to determine the virus titer and TCID50. SARS-CoV-2 was isolated from human nasopharyngeal swab confirmed positive by RT-PCR. The SARS-CoV-2 positive sample was inoculated on the 95% confluent Vero E6 cells and finally, the virus was harvested as described above in the propagation of SARS-CoV-2.

## Neutral red assay:

To determine the half-maximal inhibitory concentration (IC50) to be used for the initial assessment of chamomile for its antiviral screening, Stock solutions of the extracts in 10% DMSO with ddH2O and further diluted to the working solutions with DMEM. The cytotoxic effect of the test extracts was evaluated in Vero-E6 cells by using the previously reported Neutral Red method [26]. In brief, the cells were plated in 96-well plates and incubated for 24 h at 37°C in 5% CO2 (100 µL/well at a density of 3 x 105 cells/mL). The cells were then treated with different concentrations of chamomile extract in triplicates. After 3 days of incubation, a neutral red 0.4% (Sigma, St. Louis, MO) was added to each well and then incubated at 37°C for 4 h. The supernatant was discarded after 4 h of incubation and cell monolayers were then fixed with 100 uL/well of 5% formaldehyde, incubated at room temperature for 5 minutes. The supernatant was discarded, and cell monolayers were washed with sterile 1 x PBS. Subsequently, 100 µL of a lysis solution (5 mL sterile water, 5 mL ethanol and 100 uL of acetic acid) was added to each well. Plates were incubated for 10 minutes at room temperature with shaking. Thereafter, the absorbance of solutions was measured at 540 nm using a spectrophotometer plate reader. The IC50 of the compound was the concentration that reduces the virus-induced cytopathic effect (CPE) by 50%, relative to the virus control.

## Plaque assay:

Plaque assays for the quantification of SARS-CoV-2 were performed as previously described [25] with minor modifications. Briefly, samples were serially diluted in DMEM with 10% FBS starting from 1:10 and 1 mL from each dilution, inoculated on confluent vero E6 cell monolayers and incubated for 1 h at 37°C. Then, the inoculum was removed and overlaid with DMEM containing 0.8% agarose and incubated for 3 days at 37°C. Cells were then stained with crystal violet for 4 h at 37°C. As a result, clear plaques were distinguished from the purple monolayer. Plaques were counted to determine the viral titer as plaque forming unit (PFU)/mL. The difference between viral titer after chamomile extract treatment and untreated control was expressed as an inhibition percentage.

## Quantifying SARS-CoV-2 mRNA within infected Vero E6 cells by qPCR:

The amount of SARS-CoV-2 mRNA in the infected Vero E6 cells was determined by Real-time RT-using PowerCheK^TM^ 2019-nCoV Real-time PCR Kit (Cat No. R6900TD) in the presence and absence of the tested compound. The kit was utilized according to the manufacturer's instructions to target the RdRp gene of 2019-nCoV as previously described [26]. The ExiPrep™ 96 Viral DNA/RNA Kit (BioNEER Corp.) was used to extract the SARS-CoV-2 RNA from the infected Vero E6 cells and used in qPCR. The level of the SARV-CoV-2 mRNA was converted to cycle threshold (CT) values.

## Results & Discussion:

**Figure 1:**
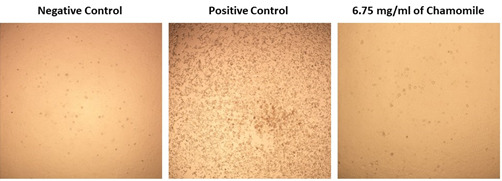
Negative control (uninfected cells), positive control (infected cells by SARS-CoV-2) and cytotoxicity (Chamomile with uninfected cells).

**Figure 2:**
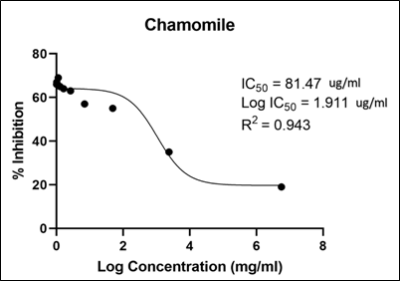
Different concentrations of chamomile extract with fixed TCID50 of SARS-CoV-2 on Vero E6 cell line. We found that the 6.75 mg/mL of chamomile extract inhibits the virus no cytopathic effect (CPE). When decreasing the chamomile extract concentration, the CPE appears gradually.

**Figure 3:**
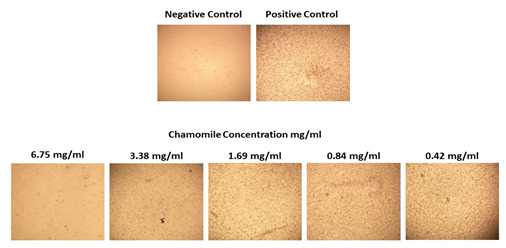
Calculation of the IC50 of chamomile extract on SARS-CoV-2. The IC50 = 81.47 ug/mL, log (IC50) = 1.911 ug/mL, and R squared (R2) = 0.943 for Chamomile extract.

Cytotoxicity and *in vitro* anti-SARS-CoV-2 assay: Despite the importance of computational analysis and molecular docking for the detection and screening of antivirals, examining the antivirals *in vitro* is a critical process that reflects the actual results on living cells similar to human cells. Here, we tested the cytotoxicity of chamomile extract on Vero E6 cell line with different concentrations. Based on the cytotoxicity result, 6.75 mg/mL chamomile extract and lower concentrations showed no CPE on Vero E6 cell line (Figure 1). Thus, 6.75 mg/mL of chamomile extract was used for anti-SARS-CoV-2 assay as this concentration considered non-toxic for Vero E6 cell line (Figure 1). The treatment protocol was conducted by applying chamomile extract and the life SRAS-CoV-2 directly to Vero E6 cell line. After three days of incubation at 37°C, 6.75 mg/mL of chamomile extract illustrated no cytopathic effect (CPE) which revealed a potential anti-SARS-CoV-2 activity of chamomile extract compared with the positive control (Figure 2). Moreover, the IC50 for chamomile extract was 81.47 ug/mL with R2 value of 0.943 (Figure 3).

## Quantification of SARS-CoV-2 mRNA within the infected Vero E6 cells by qPCR:

SARS-CoV-2 genomic titer was quantified using qPCR as described in section 2.2.4. The extracted Vero E6 cells which was infected with SARS-CoV-2 was used as a positive control in the qPCR experiment whereas, sterile Vero E6 cells was used as a negative control. Further, SARS-CoV-2 genomic titer was quantified from Vero E6 cells infected with a mix of SARS-CoV-2 and 6.75 mg/mL of chamomile extract. The CT value for the positive control was 12.3 whereas, the CT value for the cells infected with a mix of SARS-CoV-2 and 6.75 mg/mL of chamomile extract was 21.26. This means that 6.75 mg/mL of chamomile extract was able to inhibit the viral replication as the CT value was significantly higher than the value of the positive control and thus, the higher the CT value the lower the viral load which proved the potential anti-SARS-CoV-2 activity of chamomile extract.

## Plaque forming unit (PFU) result:

**Figure 4:**
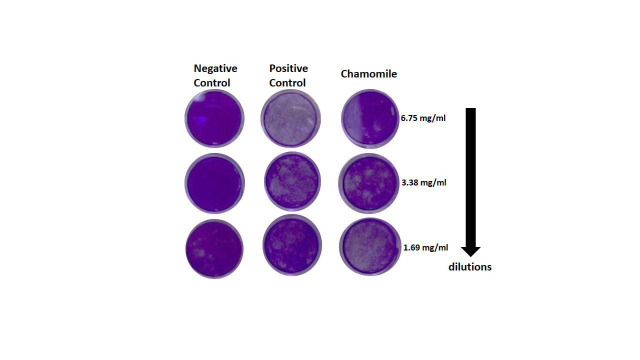
Different concentrations of chamomile extract with SARS-CoV-2 on Vero E6 cell line. We found the 6.75 mg/mL of chamomile compound inhibit virus no plaque forming unit (PFU), when decreasing the chamomile extract concentration, the plaque-forming appears gradually.

Plaque forming unit assay result is illustrated in Figure 4. Plaques were counted to determine the viral titter as plaque forming unit (PFU)/ml. The uninfected Vero E6 cells (negative control) showed undetectable replication competent of viral particles. While the positive control (Vero E6 cells infected with SRAS-CoV-2) showed detectable replication competent of viral particles after three days of inoculation (Figure 4). Compared with the positive control, there was no cytopathic effect (CPE) when incubating SRAS-CoV-2 with 6.75 mg/mL of chamomile extract for three consecutive days, which revealed the potential anti-SARS-CoV-2 activity of chamomile extract (Figure 4).

## Conclusion:

We investigated the neutralizing activity of chamomile flower decoction against SARS-CoV-2 *in vitro*. Our experiments demonstrated that chamomile could potentially have antiviral properties. Our findings suggest that further research into chamomile for the treatment of COVID-19 is warranted.
